# The Role of Nested Systems in EFL Students' Willingness to Communicate (WTC) and Engagement

**DOI:** 10.3389/fpsyg.2021.759393

**Published:** 2021-09-24

**Authors:** Lingang Gu, Pingping Sun

**Affiliations:** ^1^Special Equipment Institute, Hangzhou Vocational and Technical College, Hangzhou, China; ^2^Business & Tourism Institute, Hangzhou Vocational and Technical College, Hangzhou, China

**Keywords:** complexity dynamic system theory, willingness to communicate, student's engagement, EFL, nested system

## Abstract

Language learning is a complex process with many intrapersonal and interpersonal processes which are nested within smaller systems, themselves. Willingness to communicate (WTC) and engagement of students are two of the many complicated, multifaceted, and dynamic variables in L2 learning that have mostly been explored *via* quantitative, correlational, and one-shot methodologies. However, such a research trend provided only a snapshot of variables of second language acquisition (SLA) nature and dynamism. Against this shortcoming, this study aims to present the conceptualizations, applications, and implications of complexity dynamic system theory for investigating L2 earner-psychology variables, especially WTC and engagement. In doing so, the definitions, dimensions, and key properties of the two constructs were explained. In the end, a series of research gaps, implications, and future directions are suggested for future researchers in this territory.

## Introduction

Language learning is widely recognized as a complex process that involves various cognitive and linguistic skills to occur (Freeman and Cameron, 2008). This complexity multiplies when it is coupled with psychology, emotions, and interpersonal skills. This is because most language learning variables, especially those related to effect and psychology of learner, are highly connected and nested in other variables. The story becomes more convoluted as most language learning factors develop and fluctuate across time and contexts (MacIntyre et al., 2020). Consequently, studying language learning constructs is an arduous task to be done solely by traditional approaches. However, a bulk of existing studies on psychological traits in language learning has used quantitative and correlational designs because of their focus on generalizability (Nematizadeh and Wood, 2019). Likewise, researching willingness to communicate (WTC), which developed from the concept of unwillingness to communicate (Burgoon, 1976), has been limited to quantitative and associational techniques that perceived the construct as a fixed, trait-like feature that may not change across time and situations. This numerical orientation is observable in many scholarly works.

This simplistic outlook examined only the external correlates of WTC at the expense of its underlying, nested layers. This runs contrary to the groundbreaking study of MacIntyre et al. (1998), which unfolded the multilayered and dynamic nature of WTC for the first time. They also urged researchers to scrutinize the dynamism of many L2 constructs *via* complexity dynamic system theory (CDST). CDST is a meta-theory that highlights the underlying systems and subsystems of a phenomenon to reveal its dynamic interactions and developments (Larsen-Freeman, 2019; Amerstorfer, 2020). Based on this theory, many behaviors and events are unpredictable, dynamic, nested, and in an interactive relationship with other factors (Larsen-Freeman, 2019). These properties perfectly fit with language learning, in general, and L2 communication competencies, in particular, due to their remarkable fluidity, dynamism, and unpredictability. Another variable largely left to be examined through CDST is an engagement of the student which is multidimensional and affected by several internal and external factors (Guilloteaux, 2016). The concept concerns the degree of involvement of students in classroom activities (Skinner and Pitzer, 2012). The developmental nature of engagement sufficiently endorses the application of this new perspective.

In the pertinent literature, there exist some studies on different L2 psychology constructs such as anxiety, (de)motivation, agency, self-efficacy, identity, and enjoyment using CDST perspectives (Kaplan and Garner, 2017; Almutlaq and Etherington, 2018; Boudreau et al., 2018; Hiver and Papi, 2019; Larsen-Freeman, 2019). However, few studies (if any) have explored the constructs of WTC and engagement in English as a foreign language/English as a second language (EFL/ESL) contexts *via* this meta-theory. This justifies an urgent need for running more studies on these crucial variables in different cultural contexts using CDST methodologies to unpack their nature, developmental trajectories, and inherent dynamism. To this end, this study discusses the applications of CDST to WTC and engagement.

## Background

### Complexity Dynamic System Theory

Complexity dynamic system theory is a theory that originated from hard sciences to study change, dynamism, and evolution of complex systems and subsystems of a phenomenon (Nematizadeh and Wood, 2019; Yang, 2021). It explores the underlying processes and factors involved in the performance of a variable and its related variables that dynamically interact to cause an event (Amerstorfer, 2020). Instead of a macroscopic approach, CDST takes a holistic approach to examine complex systems by focusing and engaging in a deeper observation of the phenomenon. It also maintains that the complexity of a system increases when it comprises different, interrelated elements, and such elements, themselves, function as complex systems (Hilpert and Marchand, 2018). Hence, the interdependence and incessant coaction of constituent components of a system mark the milestone of CDST and its dynamic nature (Overton, 2015).

In this theoretical lens, events are no longer considered as predictable, fixed, isolated, and in a simple linear relationship (Larsen-Freeman, 1997). The theory suits SLA as many of its tenets are mirrored in language as a system wherein several factors continuously interact and the overall performance is the upshot of individual, interweaved elements. Based on this theory, language learning is a complex system with many other nested subsystems that reciprocally and unpredictably influence each other (de Bot et al., 2007). To put it differently, language learning is the outcome of a network of personal, cultural, social, psychological, and contextual factors which are in a coadaptive association with unclear boundaries (Mercer, 2016). Consequently, a small fluctuation in one of its underlying elements can significantly affect the performance of the whole system and the results.

### Features and Contributions of CDST

Many key properties have been proposed for CDST in the literature. However, de Bot et al. (2007) classified such features into four properties including (1) *sensitive dependence on initial conditions*, (2) *variation/change in and among elements*, (3) *the interconnectedness of subsystems*, and (4) *the presence of attractor states*. The first characteristic that is also known as *the butterfly effect* suggests that small variations in the initial (sub)system can significantly affect its subsequent performance. Therefore, tiny initial variations between two EFL students (e.g., age, proficiency) may trigger drastic variations in other aspects of their language learning journey. This substantiates the application of CDST to study WTC and engagement as the initial WTC level of a student and engagement can potentially affect his/her later academic performance. The second feature is change (variation, dynamism) which is central to CDST. It is represented *via* three other concepts of *non-linearity* (the relationship between two factors in language learning is not always linear and straightforward); *self-organization* or *reorganization* (systems and subsystems do not follow a predetermined blueprint, instead they are naturally prone to organize themselves and illustrate coherent patterns); and *idiosyncrasy* (trajectories of change and development of a system operate in distinctive manners). These reflect the uniqueness of learning and psychological variables (WTC, engagement) of the system of learners as they are unique to an individual, constantly changing their states, and in a non-linear relationship with other factors that make their measurement challenging for researchers.

*Interconnectedness* is another characteristic of CDST, which argues that dynamic systems include interconnected subsystems, which are made up of smaller subsystems that interact in their own groups and at various layers. The fourth feature is *the presence of attractor states* which refers to the tendency of a system to be in a specific state at a specific moment. This is the opposite of *repeller state* which the system avoids being in. Furthermore, there are other features for CDST in the literature as illustrated in Table 1.

**Table 1:** Additional features of CDST.

**Feature**	**Description**
Timescales	The effects of time on a process or attribute. Attributes may reveal themselves in short-run, long-run, or even moment-by-moment. Timescales are nested in each other as seconds in minutes, minutes in hours, hours in days, days in weeks, weeks in months, months in years Freeman and Cameron, 2008.
Openness	The effect of unpredicted sources on a process or construct which elucidate its dynamics and disclose its nature.
Unpredictability	Unlikelihood to predict a forthcoming event or process even by knowing the whole system and its interactions.
Stability	The relatively constant state of the overall system, for a period of time, despite the present variations.
Variability	The current state of a system is the outcome of variations to a previous state.
Emergence	The overall state of a system is greater than sum of its interacting elements.
Soft assembly	The underlying elements of a system can be re-configured into clear patterns as systems self-organize.
Fractalization	A system's ability to demonstrate and predict self-similar patterns/behaviors across different levels and timescales.

### The Concept of WTC

The concept of WTC is now an essential component of any successful L2 education that creates willing communicators. It was developed from the notion of *unwillingness to communicate* and in relation to L1 performance. This led to perceiving WTC as a fixed, trait-like, and individual-based construct. However, research approved that L2 WTC is beyond that of L1 as it has situational dynamicity and is the offshoot of an interplay of numerous sociocultural, motivational, political, identity, and pedagogical factors (MacIntyre and Legatto, 2011). By definition, WTC refers to the readiness of the interlocutor to enter into interaction by choosing to initiate the communication (MacIntyre, 2020). It is the probability that a person may start an L2 conversation without fear. Like anxiety, WTC can either be trait or situational. Trait WTC is a constant tendency to initiate a conversation, while situational WTC emerges from a specific situation (Nematizadeh and Wood, 2019).

Nowadays, the concept is regarded as dynamic and multidimensional that varies across individuals, contexts, and times after the seminal studies of MacIntyre and other leading figures. Moreover, empirical studies indicated that WTC is affected/predicted by an array of learner-psychology factors including personality, age, gender, motivation, attitudes, self-esteem, engagement, self-confidence, and cultural orientation (Saidi, 2018). These correlate variables confirm the complexity of WTC in that these subsystems can determine the decision of an individual to initiate interaction. Likewise, WTC is dynamic in that its sublayers can change, hence WTC level of an individual may increase/change, too. Therefore, the conceptualization and analysis of WTC by CDST is unquestionably plausible in EFL contexts.

### Engagement of Students: Definitions and Dimensions

Engagement of a student refers to his/her degree of involvement in classroom activities (Skinner and Pitzer, 2012). It is vital for shaping human competencies and it is a holy grail for language learning scholars (Sinatra et al., 2015). Moreover, it is a dynamic construct that is influenced by several internal and external factors (Guilloteaux, 2016). Like WTC, engagement is multidimensional and comprises behavioral, emotional, cognitive, agentic, academic, motivational, and social dimensions (DeVito, 2016). They all interact at the moment to produce engagement in students. When accomplishing a task/activity, students experience different sideline emotions, thoughts, behaviors that influence their engagement level. These components constantly change and interact with each other making the construct complex and dynamic.

Research reveals that the engagement of the student can predict different academic outcomes such as achievement, psychosocial adjustment, resilience, effective learning, and academic success (Jang et al., 2016). Furthermore, due to its flexibility, engagement is affected by different phenomenological, demographic, and instructional factors (Guilloteaux, 2016). From a CDST perspective, the dimensions and underlying components of engagement and their subcomponents can have part-to-part, part-to-whole, and whole-to-part coactions that generate the overall engagement of the student in the class (Symonds et al., 2021).

### Implications, Gaps, and Future Directions

In this mini review article, it was pinpointed that many SLA constructs including WTC and engagement are multifaceted, dynamic, and nested within their own smaller sublayers. Hence, it is feasible to apply CDST perspective to many learner-psychology variables to unfold their nature and behaviors. Consequently, this article can afford precious implications for EFL teachers, students, teacher trainers, and researchers. More particularly, the results are beneficial for EFL teachers in which they can increase knowledge and awareness of teachers of the nature and dynamism of WTC and engagement as two momentous variables in L2 education. Both variables are complicated, multilayered, and dynamic (Liu and Song, 2021). So, EFL teachers can use suitable classroom tasks which enrich WTC and classroom engagement of students. Likewise, students can use the ideas of this study in which they can identify that their language learning process is complex with numerous nested variables which have smaller sublayers. Therefore, they invest more time and effort in developing their psychological traits as preconditions for language development. The results are valuable for teacher trainers in that they can conduct workshops and training programs on the dynamic, developmental trajectories of essential variables to language learning (e.g., WTC, engagement) by teaching teachers appropriate methods and techniques that tap into the nested, complicated, and dynamic nature of psychological variables of SLA. Finally, L2 researchers can use the propositions made in this study and make attempts to locate and bridge the gaps in this area. As mentioned earlier, a huge body of research on WTC and engagement has been quantitative, correlational, and cross-sectional capturing a snapshot of the developmental process of such complicated variables. Hence, avid researchers are suggested to employ dynamically informed methodologies to uncover the complexity and dynamism of SLA constructs. Focusing on the dynamic nature of WTC, researchers can also examine the effect of different language skills on the level of WTC of EFL students. Another line of research can be conducting longitudinal research on WTC, engagement, and other SLA constructs adopting both micro and macro timescales to navigate their fluctuations since they mostly occur in a long run. Moreover, WTC can be studied in relation to different positive psychology constructs (e.g., resilience, enjoyment, optimism, love, and hope; Wang et al., 2021) as well as negative emotions such as boredom, doubt, anxiety, demotivation, and so forth. Furthermore, WTC can be scrutinized from the lens of teacher-student interpersonal communication behaviors, such as teacher care, clarity, credibility, rapport, stroke, immediacy, confirmation to see how they can impact WTC of students, engagement, success, and achievement (Xie and Derakhshan, 2021). Furthermore, WTC and the engagement of students in online education can be a striking line of inquiry. Finally, cross-cultural studies on WTC and engagement are also recommended.

## Author Contributions

Both authors have directly and substantially contributed to this manuscript and have approved its submission to Frontiers in Psychology.

## Conflict of Interest

The authors declare that the research was conducted in the absence of any commercial or financial relationships that could be construed as a potential conflict of interest.

## Publisher's Note

All claims expressed in this article are solely those of the authors and do not necessarily represent those of their affiliated organizations, or those of the publisher, the editors and the reviewers. Any product that may be evaluated in this article, or claim that may be made by its manufacturer, is not guaranteed or endorsed by the publisher.
